# Attachment and reflective functioning in families with a child on the autism spectrum

**DOI:** 10.3389/fpsyg.2025.1651408

**Published:** 2025-11-03

**Authors:** Charlotte Engberg Conrad, Carol George, Emil Færk, Helle Jakobsen, Per Hove Thomsen, Marlene Briciet Lauritsen

**Affiliations:** ^1^Department of Psychiatry, Aalborg University Hospital, Aalborg, Denmark; ^2^Department of Psychology, Mills College at Northeastern University, Oakland, CA, United States; ^3^Department of Child and Adolescent Psychiatry, Aarhus University Hospital, Psychiatry, Aarhus, Denmark; ^4^Department of Clinical Medicine, Aarhus University, Aarhus, Denmark; ^5^Department of Clinical Medicine, Aalborg University, Gistrup, Denmark

**Keywords:** autism, attachment, adult attachment, parental attachment, reflective functioning, parental reflective functioning, child attachment

## Abstract

The concepts of attachment and reflective functioning are predictors of positive development in children on the autism spectrum. This is the one of the first cross-sectional studies to examine associations between parents’ attachment representations and parental reflective functioning and child attachment in families with children on the autism spectrum. Twenty-eight parents completed the Adult Attachment Projective Picture System and questionnaires of Maternal Perception of Child Attachment and Parental Reflective Functioning regarding their child on the autism spectrum and when applicable a typically developing sibling. To test any associations between the parents’ attachment and parental reflective functioning and parents’ perception of child attachment, the sample was divided in organized (secure, dismissing and preoccupied combined) as compared with unresolved parents. We found a higher level of the parents’ Interest and Curiosity in their child’s mental states (a parental reflective functioning domain) in the organized as compared with unresolved parents. Also, mothers had significantly higher levels of Interest and Curiosity than fathers. There were no other significant differences regarding the remaining questionnaire domains. Also, there were no significant differences between parents’ rating of child attachment or parental reflective functioning in relation to their child on the autism spectrum compared with their typically developing sibling. The findings suggest that future support may enhance focus on parents unresolved to loss and trauma and fathers. Also, more research is needed to understand the implications of attachment and reflective functioning in families affected by autism.

## 1 Introduction

Autism is a pervasive neurodevelopmental disorder characterized by difficulties in social communication, and repetitive stereotyped behaviors and interests (WHO, 2024). According to attachment theory, the development of secure internal working models is beneficial for healthy emotional, social and cognitive development (Bosmans and Borelli, 2022; Dagan et al., 2024; Schore, 2001). Intergenerational transmission of attachment patterns is related to parents’ attachment representation and parental reflective functioning (Luyten et al., 2017b; van IJzendoorn, 1995; van Ijzendoorn and Bakermans-Kranenburg, 2019). These processes are challenged by a child’s autism characteristics, as the ability to understand, predict, and affect the caregiver’s behavior may be affected by difficulties in these children’s social communication (Cortina and Liotti, 2010; Stern, 2004; Teague et al., 2017). The current cross-sectional study examines the relation between parent’s attachment representations and their perception of the child’s attachment and parental reflective functioning.

Attachment is an innate neurobiological behavioral system providing the child with a fundamental sense of security through the perceived availability of their primary caregiver (i.e., attachment figure) when the child is stressed (Bowlby, 1969). Attachment bonds are developed in the interaction between the caregiver and child through mutual and recognizable dynamic reciprocal communicative interactions, where positive and negative interactions will affect parent and child behavior (Ainsworth et al., 1978). The child will develop a representational model of self, other, and relationships that becomes a lifelong interpretation of how to understand and interpret experiences and emotions (Bowlby, 1969, 1973).

Children on the autism spectrum have the same ability to form attachment bonds as typically developing children (Kahane and El-Tahir, 2015; Rutgers et al., 2004; Teague et al., 2017). One longitudinal study shows that children on the autism spectrum classified secure demonstrate better language development and higher empathic ability later in childhood than insecure children (Rozga et al., 2018). This study also demonstrates the positive sequelae of secure attachment are comparable to a normative population (Rozga et al., 2018). Other studies have indicated that children with autism show signs of more insecurity towards their parents than typically developing children (Rutgers et al., 2004; Teague et al., 2017). These studies suggest that this is due to the innate social communication disabilities seen in children on the autism spectrum. A study by Teague et al. (2018) found a higher frequency of insecure attachment in children on the autism spectrum than in samples of typically developing children, and insecure attachment was related to the child’s diagnosis, cognitive development, and parenting practices (e.g., coercive parenting and closeness in the attachment relationship) (Teague et al., 2018). This finding underscores the dynamic, bi-directional interplay involved in the development of attachment the quality (Sameroff, 2009; Teague et al., 2018).

As early as 1940 John Bowlby emphasized the significance of the transference of patterns between parents and their children. He thought that supporting the parents could help the child’s development (Bowlby, 1940). Since then, attachment research has demonstrated that parents’ attachment representation to some extent is transferred to the child (Benoit and Parker, 1994; Buchheim et al., 2022; Fonagy et al., 1991a; George and Solomon, 1996; Solomon and George, 2011; van IJzendoorn, 1995; van IJzendoorn and Bakermans-Kranenburg, 1997). This correlation has not been sufficiently investigated concerning children on the autism spectrum (Teague et al., 2017).

Attachment patterns in parents of children on the autism spectrum have only been examined in a few studies using developmental assessments of adult attachment (Bond et al., 2020; Conrad et al., 2025; Seskin et al., 2010; Teague et al., 2017). The studies are small and have conflicting results. The study by Seskin et al. (2010) showed a similar distribution of adult attachment in 40 parents of children on the autism spectrum as the distribution pattern found in normative samples as measured by the Adult Attachment Interview (AAI). The study by Seskin et al. (2010) found that children whose parent’s adult attachment is secure had better relational and functional abilities than children of insecure parents (Seskin et al., 2010). A case–control study by Bond et al. (2020) found insecure adult attachment representations in all four participating parents as measured by the AAI. Conrad et al. (2025) examined adult attachment in a sample of 37 parents of children on the autism spectrum and found that 34 of them were classified as insecure, based on the Adult Attachment Projective Picture System (AAP) (Conrad et al., 2025).

Parental reflective functioning (PRF) refers to parents’ mentalizing capacities to reflect upon their own internal mental experiences as well as those of the child (Fonagy et al., 1991b; Luyten et al., 2017a). PRF is believed to be important in the development of the child’s own mentalizing capacity and secure attachment (Kelly et al., 2005; Sharp and Fonagy, 2008; Slade, 2005). A previous review emphasizes the need for research on the impact of reflective functioning in families affected by autism. This knowledge will build our understanding of intergenerational transmission of attachment (Teague et al., 2017). The social communication disabilities of children on the autism spectrum may complicate parent’s ability to comprehend children’s mental states and motivations. As a result, the parent’s behavior may be difficult for the child to predict and understand leading to interactions reflected in lower levels of PRF (Slade, 2009; Stern, 2004; Teague et al., 2017). However, parents who can adapt to their children’s development and make more of an effort in the understanding of their child would be expected to lead to higher levels of PRF. Not all children in a family are the same, so it seems possible that parenting would be different for typically developing siblings compared with siblings on the autism spectrum. Previous studies found that typically developing siblings receive less parental attention and differential treatment than their siblings on the autism spectrum (Chan and Goh, 2014; Enav et al., 2020). Only one previous study by Enav et al. (2020) has examined parental reflective functioning examining children on the autism spectrum compared to typically developing siblings. This study found, in a sample of 30 parents, that parents exhibited significantly higher reflective functioning when interacting with their child on the autism spectrum than with the typically developing sibling (Enav et al., 2020).

Traditionally the primary caregiver in a family is the mother, however, this pattern is no longer true in families in Western culture. Fathers today are more active and engaged in childcare than in the past. Interest in children’s attachment to fathers is not recent (Grossmann and Grossmann, 2020). One study by Miljkovitch et al. (2004) assessed attachment in 31 children using the Attachment Story Completion Task and in both parents with the AAI. The study found a significant association of attachment between the mothers and their children, but not between the fathers and their children (Miljkovitch et al., 2004), contrary to the results of a few other studies showing concordance between the fathers’ AAI and their child’s attachment as measured by the Strange Situation Procedure (Radojevic, 1992; Steele et al., 1996; van IJzendoorn, 1995). More research is needed to understand the impact of both parents’ attachment representations on the child.

### 1.1 Purpose

The purpose of the study is to increase knowledge about the impact of attachment and reflective functioning in families with a child on the autism spectrum building on data on the classification of parents’ adult attachment and parental reflective functioning and parents’ perception of child attachment (Conrad et al., 2025).

This cross-sectional study is one of the first to examine associations between parents’ attachment representations and parental reflective function and child attachment in families with children on the autism spectrum. The study aims to examine:

1. The associations between parents’ adult attachment and parental reflective functioning.
2. The association between parents’ adult attachment and parental perception of child attachment.
3. Differences between the fathers’ and mothers’ perception of child attachment or parental reflective functioning.
4. Differences between parents’ perception of their child’s attachment and parental reflective functioning in the children on the autism spectrum compared to their typically developing siblings.

## 2 Materials and methods

### 2.1 Participants

Participants were 37 parents of 24 children on the autism spectrum and their 11 typically developing siblings partly recruited from a Danish feasibility study of the Paediatric Autism Communication Therapy intervention and partly from two Danish child and adolescent psychiatric departments. Parents received verbal and written information about the project before providing consent for their own and their children’s participation in this study. Twenty boys and four girls were included with a mean age of 5.1 years (range 3.4–7.0 years). All children were diagnosed with the following ICD-10 diagnoses: F84.0, F84.1 or F84.5, which in this study is defined as autism spectrum disorder. All participating parents were biological parents. All but three parents were cohabiting with the other parent. Only three participants were single parents. Participants were mainly educated (equally distributed representation of short, middle and long educations), employed, and from middle to high-income households. The 11 typically developing siblings were all under the age of 7 years. For further demographics see Table 1 in Conrad et al. (2025).

Parents participated in the AAP test and were asked to complete the questionnaires Maternal Perception of Child Attachment (MPCA), and Parental Reflective Functioning Questionnaire (PRFQ). The questionnaires were completed by 28 parents, 18 mothers and 10 fathers. Eleven of the parents (7 mothers, 4 fathers) also completed these questionnaires regarding a typically developing sibling under the age of 7.

### 2.2 Measures

#### 2.2.1 Adult attachment projective picture system

The AAP is a validated free response test used to designate four standard adult attachment patterns: secure, dismissing, preoccupied, and unresolved (George and West, 2011). The AAP consists of a series of pictures, which increasingly activate attachment-related distress. Parents are presented one picture at a time and are asked to describe a hypothetical situation about what is happening, what lead up to the scene, what the persons are thinking or feeling, and what will happen next. In this study all AAPs were administered virtually, which is a valid administration method (David et al., 2022). The parents’ responses were audio-recorded and transcribed verbatim. The transcripts were coded according to the AAP manual standards by the first author who is a reliable judge. Twenty percent of the transcripts were double-coded for reliability by another reliable judge with 100% agreement on attachment classification (Conrad et al., 2025).

#### 2.2.2 Maternal perception of child attachment

Child attachment was assessed using the Maternal Perceptions of Child Attachment (MPCA) parent report questionnaire (Hoppes and Harris, 1990). The MPCA has 23 items rated on a 5-point Likert Scale, with higher scores reflecting parental perception of a more secure attachment. Questions address how much the parents see the child’s either verbal or physical interactions, how the child identifies with the parent and is capable of sharing intimacy (Goodman and Glenwick, 2012). The parents’ ratings are considered a reflection of the relative strength of a child’s attachment security. The questionnaire was translated into Danish in 2021 by the first author and the translation was checked using back-translation to English by a professional translation company. The Danish version was named Parental Perception of Child Attachment because of its use with mothers and fathers. No other adaptions were made. The MPCA has previously been used and validated in populations with children on the autism spectrum (Goodman and Glenwick, 2012; Siller et al., 2014). Cronbach’s alpha in our sample showed a high internal consistency with a reliability coefficient of 0.86.

#### 2.2.3 Parental reflective functioning questionnaire

Parents’ reflective functioning about the child is assessed with the multi-dimensional, well-validated questionnaire PRFQ (Luyten et al., 2017a). The questionnaire consists of 18 items rated on a 7-point Likert Scale. It evaluates the dimensions of Pre-Mentalizing (PM), Certainty about Mental States (CMS), and Interest and Curiosity (IC). PM modes capture a non-mentalizing stance, malevolent attributions and the parent’s inability to enter the child’s world. PM includes six items, for example, “My child cries around strangers to embarrass me.” Higher scores indicate a developmentally lower level of mentalizing. CMS refers to the recognition of the opacity of mental states. This dimension has six items, for example, “I can always predict what my child will do.” Higher scores indicate a higher level of CMS. Low scores indicate hypomentalization, with a lack of certainty about the child’s mental states. IC refers to the parent’s interest in the mental states of the child. There are six items, for example, “I try to see situations through the eyes of my child.” Higher scores indicate a higher capacity of IC that reflects hypermentalizing with lower scores reflecting an absence of interest in the mental states of the child (Luyten et al., 2017a). The questionnaire was translated into Danish in 2015 by Mette Skovgaard Væver and Johanne Smith-Nielsen, without additional adaptions from the original. The factor structure of the PRFQ has been investigated in a Danish sample of 423 mothers with and without postpartum depression, where the three-factor structure of the questionnaire was supported (Wendelboe et al., 2021). Recently, the PRFQ was validated in a Finish sample of 355 mothers and 108 fathers finding factor structure similar to the original PRFQ and thus supporting cross-cultural validity (Flykt et al., 2025).

PRFQ has primarily been validated in samples of young children, and the use is only recommended in children up to the age of 5 (Luyten et al., 2017a). We decided to use the measure in the sample of children aged 2–6 years as the developmental age in children with autism is often not equivalent to the chronological age (Pastor-Cerezuela et al., 2016; Werner et al., 2005). Research shows that children on the autism spectrum exhibit delays in the development of language and adaptive functioning (Brignell et al., 2018; Jain et al., 2025). Also, PRFQ has been used for 6-year-olds in other studies (Kungl et al., 2024). Cronbach’s alpha in our sample for the PRFQ showed both low and high internal consistencies with reliability coefficients of PM 0.12, CMS 0.87, and IC 0.63.

### 2.3 Statistical analysis

Within the sample differences related to the adult attachment are analyzed with *t*-tests, when data is normally distributed. Because of the data representing both parents’ rating of their relationship with the same child, we used a mixed effects logistic regression to control for data non-independency. Correlations for parental perception of child attachment and parental reflective functioning were performed by separating parents into two adult attachment groups – secure/insecure or organized/unresolved. The level of statistical significance was for all analyses 0.05. All statistical analysis was performed using Stata Statistical Software: Release 18. College Station, TX: StataCorp LLC.

### 2.4 Ethical considerations

The study was reviewed by the local ethics committee which, according to Danish regulations, decided that no approval for the feasibility study was required. All data were following the European Union regulations, i.e., the General Data Protection Regulation. The study was registered at the regional research administration in the North Denmark Region (ID number F2022-050). Complete anonymity was ensured for the participants. Participation was voluntary, and participating parents were informed that their participation did not have any consequences for the treatment of their child. All participating parents signed an informed consent form.

## 3 Results

Using the AAP, the 37 parents were classified as 8.1% (3) *secure*, 27% (10) *dismissing*, 18.9% (7) *preoccupied,* and 45.9% (17) *unresolved* (Conrad et al., 2025). The 28 parents who filled in the PRFQ and MPCA questionnaires were classified as 7.1% (2) secure, 32.1% (9) dismissing, 17.9% (5) preoccupied and 42.9% (12) unresolved. Due to the low sample size, the low frequency of secure and the high frequency of unresolved, the sample was dichotomized in the organized (secure, dismissing, and preoccupied combined) and the unresolved for the following analyses. This dichotomization of attachment classifications is consistent with previous research (Dagan et al., 2018; Eilert and Buchheim, 2023).

### 3.1 Association between adult attachment and parental reflective functioning

t-test analyses showed no significant difference between the PM and CMS domains between the 16 organized versus the 12 unresolved parents (Table 1), or the mixed effects regression (Table 2). Regarding the PRFQ IC, the t-test found a small significant difference between parent groups: unresolved parents had a lower mean on the IC dimension than organized parents with a mean difference of −0.60 *p* = 0.05, confidence interval slightly crossing the zero [−1.21;0.01] (Table 1). The mixed effects regression coefficient of 0.60 regarding IC found a difference between the groups of organized compared to unresolved was significant *p* < 0.05 (Table 2).

**Table 1:** Two sample *t*-tests of comparing organized and unresolved parents’ ratings of their child on the autism spectrum.

	Range	Organized (*N* = 16)		Unresolved (*N* = 12)		Difference	SE	95% CI	*p*-value
		Mean	SD	Mean	SD	Mean Diff			
PRFQ PM	0.5–2.7	1.91	0.45	1.68	0.53	−0.23	0.19	−0.61;0.16	0.23
PRFQ CMS	1.7–5.8	3.90	1.26	3.74	0.92	−0.15	0.43	−1.04;0.73	0.71
PRFQ IC	4.2–7.0	5.91	0.80	5.31	0.75	−0.60	0.30	−1.21;0.01	0.05
MPCA	2.3–3.6	2.93	0.38	2.82	0.32	−0.11	0.14	−0.39;0.14	0.42

**Table 2:** Mixed effects regression of organized and unresolved parents’ ratings of their child on the autism spectrum.

Outcome	Coefficient	SE	*p*-value	95% CI
PRFQ PM	0.23	0.17	0.19	0.11;0.57
PRFQ CMS	0.16	0.42	0.70	−0.66;0.97
PRFQ IC	0.60*	0.29	0.04*	0.04;1.16
MPCA	0.13	0.13	0.34	−0.13;0.38

^*^p < 0.05.

### 3.2 Associations between adult attachment and parents’ perception of child attachment

t-test analyses found no significant differences between organized and unresolved parents’ perceptions of child attachment as measured by the MPCA (Table 1), and no significant differences were found using the mixed effects regression (Table 2).

### 3.3 Differences between fathers and mothers

Mother versus father differences were examined using mixed effects regression for both the MPCA and the PRFQ. There were no significant differences between 18 mothers’ and 10 fathers’ ratings for the PRFQ PM, PRFQ CMS and MPCA (Supplementary material 1).

Regarding the PRFQ IC, we found evidence of mothers experiencing a significantly higher interest and curiosity in their children’s mental states compared to the fathers with a mixed effects coefficient 0.64, 95% CI [0.06, 1.21], *p* < 0.05 (Supplementary material 1).

### 3.4 Siblings

Differences between the groups of organized and unresolved parents were also tested regarding the 11 typically developing siblings. None of the included PRFQ and MPCA outcomes showed any significant differences between the groups (Table 3).

**Table 3:** Two sample *t*-tests of parents rating of typically developing siblings.

	Organized (*N* = 8)		Unresolved (*N* = 3)		Difference	SE	CI	*p*-value
	Mean	SD	Mean	SD	Mean Diff			
PRFQ PM	1.42	0.53	1.17	0.17	−0.25	0.32	−0.98;0.48	0.46
PRFQ CMS	4.40	1.17	3.72	0.25	−0.67	0.71	−2.27;0.92	0.37
PRFQ IC	6.00	0.82	5.22	0.84	−0.78	0.56	−2.04;0.49	0.20
MPCA	2.60	0.19	2.70	0.26	0.10	0.14	−0.22;0.41	0.50

The 11 typically developing siblings were compared to their 11 siblings on the autism spectrum. No significant differences were found in this small sample (Table 4).

**Table 4:** Comparison between siblings.

	Children on the autism spectrum (*N* = 11)		Typically developing siblings (*N* = 11)		Difference	SE	CI	*p*-value
	Mean	SD	Mean	SD	Mean Diff			
PRFQ PM	1.58	0.29	1.35	0.58	0.23	0.17	−0.16;0.62	0.22
PRFQ CMS	3.68	1.19	4.21	1.04	−0.53	0.31	−1.23;0.17	0.12
PRFQ IC	5.73	0.93	5.79	0.86	−0.06	0.12	−2.04;0.49	0.20
MPCA	2.72	0.39	2.62	0.20	0.10	0.12	−0.22;0.41	0.50

## 4 Discussion

The purpose of this study was to advance our understanding of attachment and PRF in families raising a child on the autism spectrum. Regarding PRF findings indicated that parents with organized attachment patterns reported significantly higher levels of Interest and Curiosity (IC) compared to those with unresolved attachment patterns. This suggests that organized parents demonstrate a greater capacity for reflective engagement with their child’s mental and emotional states. The IC domain reflects the degree to which parents express thoughtful consideration of their child’s internal experiences, as captured by items such as “I wonder a lot about what my child is thinking and feeling” and reverse-scored items like “I believe there is no point in trying to guess what my child feels.” Although parents with organized attachment also showed slightly higher scores on the PM and CMS domains, these differences were not statistically significant.

The results indicated that organized and unresolved parents reported similar perceptions of their children’s attachment. This may be expected, as only two of the 28 parents who completed the questionnaires were classified as secure. Consequently, the organized group consisted primarily of insecurely attached parents (preoccupied or dismissing), and unresolved attachment also reflects an insecure pattern.

The mean MPCA score of 2.72, indicating lower child attachment security, was comparable to the baseline mean of 3.06 reported by Siller et al. (2014). This lower perceived attachment security in our sample is likely related to the high prevalence of insecure parental attachment.

Comparing mothers and fathers, mothers reported higher PRFQ IC scores than fathers. In our sample, more fathers were classified unresolved, which may partly explain this difference. The finding aligns with previous studies finding higher IC levels in mothers compared to in non-clinical samples (Flykt et al., 2025; Luyten et al., 2017a). However, we did not observe a significant difference in CMS, possibly due to our low sample size.

Regarding the parents’ report of MPCA and PRFQ for their typically developing child, no significant differences were found between the groups of organized and unresolved parents. A similar trend of higher IC levels organized parents, with a group difference of 0.78, was observed but did not reach significance due to the small sample size of 11 siblings.

There were no significant differences in parents’ MPCA or PRFQ ratings between their children on the autism spectrum compared to their typically developing siblings. This contrasts with Enav et al. (2020), who found higher PRF ratings for children on the autism spectrum than the typically developing sibling. The discrepancy may reflect the complex dynamics of parental reflective functioning in these families. Parents may invest greater effort and persistence in understanding their child on the autism spectrum, thus enhancing their parental reflective functioning. At the same time, the child’s social-communicative challenges may hinder parental understanding, limiting reflective capacity. These opposing factors may co-exist, balancing out potential differences in parental reflective functioning between children on the autism spectrum and their typically developing siblings.

### 4.1 Strengths and limitations

This study was one of the first to examine associations between parents’ attachment representations and parental reflective function and child attachment in families with children on the autism spectrum. Validated measures of attachment and parental reflective functioning were used in this study.

The primary limitation of the study was the small sample size and missing data on the PRFQ and MPCA questionnaires, resulting in limited statistical power. Nonetheless, it is expected the findings in future meta-analysis will contribute to a growing evidence on the examined associations. Another limitation was the sample’s predominance of parental attachment insecurity. Given that defensive processes linked to preoccupied and dismissing attachment support organization of the attachment system (George and West, 2012), combining these groups with secure parents was justified, and consistent with previous research (Dagan et al., 2018; Eilert and Buchheim, 2023). However, with a larger, more diverse sample, we would have preferred to analyze secure versus insecure attachment or the four attachment styles separately.

Defensive processes are known to influence how individuals respond to self-report measures (George and West, 1999). Self-report questionnaires for parents of children with autism may be especially stressful because endorsing items evaluating the self or their child may cause feelings of inadequacy, harshness, or guilt, or bad conscience about their typically developing child. Studies comparing social psychology self-report measures of attachment and developmental measures show that individuals inflate self-reported security (George and West, 1999). This bias may similarly influence PRFQ scores. Potential limitations of the PRFQ include its brevity, its design as a screening tool for large samples (Luyten et al., 2017a) and its retrospective nature. Observational methods or parental interviews are generally considered more accurate (Luyten et al., 2017a). We recommend that future research in attachment and parental reflective functioning should incorporate observational data or semi-structured interviews to validate any self-report findings.

## 5 Conclusion

Our findings indicate that parents do not perceive significant differences in attachment-related behaviors between their child on the autism spectrum and their typically developing sibling. However, unresolved parents and fathers of children on the autism spectrum showed slightly greater difficulties regarding the PRFQ domain IC. Professionals working with these families may consider providing targeted support to enhance this aspect of parental reflective functioning. This could be by offering parent-mediated interventions that strengthen parental reflective functioning. Further research with larger and more diverse samples is needed to deepen our understanding of the interplay between attachment and parental reflective functioning in families affected by autism.

## Data Availability

The raw data supporting the conclusions of this article will be made available by the authors, without undue reservation.
