# Supplementary material for: Attachment and reflective functioning in families with a child on the autism spectrum
Source: Front Psychol. 2025 Nov 3;16:1651408. doi: 10.3389/fpsyg.2025.1651408 (PMC12620248; doi:10.3389/fpsyg.2025.1651408)
Supplement: Supplementary file 1 [file Table_1.docx]

**Supplementary material 1.** Mixed effects regressions models for PRFQ and MPCA in mothers and fathers

| Various mixed regression models with PRFQ and MPCA as outcome and parents as input, controlled for attachment. The family structure is taken into account in the random effect. (N=28, Families=20) | | | | | | |
| --- | --- | --- | --- | --- | --- | --- |
|  | | Parent gender as input: Mother (reference=Father) | | | | |
| Output measure in each model: | | Coefficient | SE | p | 95% CI | |
|  | PRFQ Certainty about Mental States | -0.47 | 0.46 | 0.314 | -1.37 | 0.44 |
|  | PRFQ Interest and Curiosity | 0.64* | 0.29 | 0.030* | 0.06 | 1.21 |
|  | PRFQ Pre-Mentalizing | 0.01 | 0.16 | 0.958 | - 0.31 | 0.32 |
|  | MPCA | - 0.02 | 0.14 | 0.884 | - 0.30 | 0.25 |

**p* < 0.05
